# Supplementary material for: Quantum key distribution implemented with d-level time-bin entangled photons
Source: Nat Commun. 2025 Jan 2;16:171. doi: 10.1038/s41467-024-55345-0 (PMC11696242; doi:10.1038/s41467-024-55345-0)
Supplement: Supplementary file 1 — Supplementary Information [file 41467_2024_55345_MOESM1_ESM.pdf]

Quantum key distribution implemented with *d*-level time-bin entangled photons

Supplementary Information

Supplementary Note 1 - Experimental details

Supplementary Note 1.1 - Experimental scheme for quantum state processing

The setup used for quantum state processing and phase-basis measurement in the QKD experiment had a total transmission loss of ~17.5 dB, which included ~5 dB for the programmable filter, ~3 dB for the phase modulator, ~7.5 dB for the on-chip interferometer cascade (OIC, 4.5 dB insertion loss and 3 dB splitting loss), and ~2 dB for the fiber components (polarization controllers and polarization beam splitters, used for polarization alignment). Phases to qudit states were applied through external phase modulation, which was implemented by using the programmable filter, the electro-optic phase modulator, and the arbitrary waveform generator. First, we optimized the system by making the classical pump burst interfere, and second, we processed the generated photonic qudits. Both classical and quantum interference measurements could be achieved without changing the setup, by simply adjusting the spectral bands of the programmable filter. For system optimization, the programmable filter was set in such a way to let pass the pump pulses toward the phase modulator and the OIC. This way, we could straightforwardly align the timing between electronic and optical signals through monitoring the interference of the temporally delayed classical pump pulses using an ultrafast photodiode (50 GHz bandwidth) and an electronic sampling scope (Tektronix, CSA8200, 76 GHz bandwidth). Upon successful alignment, we then switched the programmable filter to block the pump's band and let pass the signal and idler photons' bands only, for quantum state processing. We note that future OIC designs could involve active, thermal phase controls on the unbalanced Mach-Zehnder interferometers (MZIs) to ensure full quantum state processing within the OIC without external phase modulation. This would allow removing the electro-optic phase modulators, the programmable filter, and some of the fiber components, thus reducing the total losses of the setup by ~9 dB, with the overall loss of the processing system at ~8.5 dB.

Supplementary Note 1.2 - Operational scheme of the on-chip interferometer cascade

The on-chip interferometer cascade (OIC) consists of a fully connected concatenation of balanced and unbalanced (MZIs, see Supplementary Figure 2). The transmission matrix for a single 50:50 coupler is

$$\hat{T}_{50:50} = \frac{1}{\sqrt{2}}\begin{pmatrix} 1 & i \\ i & 1 \end{pmatrix}. \quad (1)$$

The transmission matrix for all j balanced MZIs, considering the relative phase between two arms $\Delta\psi_j$, can be expressed as

$$\hat{T}_{\mathrm{MZI}}^{(j)} = \hat{T}_{50:50}\begin{pmatrix} e^{i\Delta\psi_j} & 0 \\ 0 & 1 \end{pmatrix}\hat{T}_{50:50} = \frac{1}{2}\begin{pmatrix} e^{i\Delta\psi_j} - 1 & ie^{i\Delta\psi_j} + i \\ ie^{i\Delta\psi_J} + i & 1 - e^{i\Delta\psi_j} \end{pmatrix}, \quad (2)$$

with $j = 1, 2, \dots, 10$. The output splitting ratio of each MZI can be tuned by controlling the voltage of electric heaters on top of the waveguide, thus changing the relative phases $\Delta\psi_j$. In our work, we used only three splitting ratios, i.e., full transmission (t), full reflection (r), and 50:50 splitting, which correspond to the relative phases $\Delta\psi_j = 0, \pi,$ and $\pi/2$, respectively. From this, the respective transmission matrices $\hat{T}_{MZI}^{(j)}$ reduce to

$$\begin{aligned} \hat{T}_{\mathrm{MZI}}^{(\mathrm{t})} &= \begin{pmatrix} 0 & i \\ i & 0 \end{pmatrix}, \\ \hat{T}_{\mathrm{MZI}}^{(r)} &= \begin{pmatrix} -1 & 0 \\ 0 & 1 \end{pmatrix}, \\ \hat{T}_{\mathrm{MZI}}^{(50:50)} &= \frac{1}{2}\begin{pmatrix} i-1 & i-1 \\ i-1 & 1-i \end{pmatrix} \rightarrow \frac{1}{2}\begin{pmatrix} 1 & 1 \\ 1 & -1 \end{pmatrix}, \end{aligned} \quad (3)$$

(the global phase factor $(i-1)$ in front of the matrix $\hat{T}_{\mathrm{MZI}}^{(50:50)}$ can be omitted).

The unbalanced MZIs have a relative path delay that incrementally doubles (e.g., from 1 to 2 ps, then to 4 ps, continuing up to 128 ps), thus allowing for pulse shaping on time scales beyond the capabilities of current spectral wave-shaping technologies[1]. After experiencing one unbalanced MZI delay, an input optical pulse is split to obtain two output pulses. If the coherence time of the pulse is shorter than the temporal delay between the MZIs, we can assume that there is no temporal overlap between the created pulses after they experience splitting, delay, and recombination.

The mathematical reasoning that describes the processing of generated photonic time modes through our OIC is as follows. A time mode going through a short path remains unchanged, while a time mode going through a long path experiences a temporal shift. Here, we define the operator $\hat{P}_m$ for an mth-step time shift as:

$$\hat{P}_m \hat{a}_k^\dagger |vac\rangle = \hat{a}_{k+m}^\dagger |vac\rangle, \tag{4}$$

where $\hat{a}_k^\dagger$ denotes the creation operator in the mode k, while $|vac\rangle$ is the vacuum state.

As an example of this, we consider the preparation of 4-level (ququart) entangled qudits. In this case, we activated two unbalanced MZIs having arm differences of 128 ps and 64 ps, the delay of which is represented by the shift operators $\hat{P}_2$ and $\hat{P}_1$, respectively. This configuration gives rise to a time mode spacing of 64 ps. The time-mode shift operation and the additional phase difference ($\Delta\varphi_n^{(\prime)}$) given by the two dissimilar paths of the MZIs result in the delay transmission matrices

$$\hat{T}_{128\text{ps}}^{(\text{delay})} = \begin{pmatrix} \hat{P}_2 e^{i\Delta\varphi_1} & 0 \\ 0 & e^{i\Delta\varphi_1'} \end{pmatrix}, \tag{5}$$

and

$$\hat{T}_{64\text{ps}}^{(\text{delay})} = \begin{pmatrix} \hat{P}_1 e^{i\Delta\varphi_2} & 0 \\ 0 & e^{i\Delta\varphi_2'} \end{pmatrix}. \tag{6}$$

When setting the optical switches 7, 8, and 9 to 50:50 (see Supplementary Figure 2) and the others to full transmission (t) or full reflection (r), the single time mode $\hat{a}_0^\dagger|vac\rangle$ entering the input port 2 (In_2) experiences a splitting given by

$$\begin{aligned} &\hat{T}_{\text{MZI}}^{(50:50)} \hat{T}_{64\text{ps}}^{(\text{delay})} \hat{T}_{\text{MZI}}^{(50:50)} \hat{T}_{128\text{ps}}^{(\text{delay})} \hat{T}_{\text{MZI}}^{(50:50)} \begin{pmatrix} 0 \\ \hat{a}_0^\dagger |vac\rangle \end{pmatrix} \\ &\rightarrow \frac{1}{2} \begin{pmatrix} \{e^{i(\Delta\varphi_1+\Delta\varphi_2)}\hat{a}_3^\dagger + e^{i(\Delta\varphi_1+\Delta\varphi_2')}\hat{a}_2^\dagger - e^{i(\Delta\varphi_2+\Delta\varphi_1')}\hat{a}_1^\dagger + e^{i(\Delta\varphi_1'+\Delta\varphi_2')}\hat{a}_0^\dagger\}|vac\rangle \\ \{e^{i(\Delta\varphi_1+\Delta\varphi_2)}\hat{a}_3^\dagger - e^{i(\Delta\varphi_1+\Delta\varphi_2')}\hat{a}_2^\dagger - e^{i(\Delta\varphi_2+\Delta\varphi_1')}\hat{a}_1^\dagger - e^{i(\Delta\varphi_1'+\Delta\varphi_2')}\hat{a}_0^\dagger\}|vac\rangle \end{pmatrix}. \end{aligned} \tag{7}$$

Here, we neglected the additional global phase given by other switches (couplers) and delays. The output states from ports Out_1 and $\text{Out}_{2\text{a}}$ are thus given by

$$|\Psi_1\rangle = \frac{1}{2}\left(e^{i(\Delta\varphi_1+\Delta\varphi_2)}\hat{a}_3^\dagger + e^{i(\Delta\varphi_1+\Delta\varphi_2')}\hat{a}_2^\dagger - e^{i(\Delta\varphi_2+\Delta\varphi_1')}\hat{a}_1^\dagger + e^{i(\Delta\varphi_1'+\Delta\varphi_2')}\hat{a}_0^\dagger\right)|vac\rangle \tag{8}$$

and

$$|\Psi_2\rangle = \frac{1}{2}\left(e^{i(\Delta\varphi_1+\Delta\varphi_2)}\hat{a}_3^\dagger - e^{i(\Delta\varphi_1+\Delta\varphi_2')}\hat{a}_2^\dagger - e^{i(\Delta\varphi_2+\Delta\varphi_1')}\hat{a}_1^\dagger - e^{i(\Delta\varphi_1'+\Delta\varphi_2')}\hat{a}_0^\dagger\right)|vac\rangle, \tag{9}$$

respectively. The state $|\Psi_1\rangle$ from port $\mathrm{Out_1}$ is taken to prepare time-bin entanglement (i.e., optical amplification and injection into the spiral waveguide), while the state $|\Psi_2\rangle$ from port $\mathrm{Out_{2a}}$ is blocked. Port $\mathrm{Out_{2a}}$ is then used to send the generated signal and idler photons back to the same OIC for quantum state processing. The state vector representing the signal/idler photons entering port $\mathrm{Out_{2a}}$ can be expressed as

$$\begin{pmatrix} 0 \\ \frac{1}{2}\{e^{i(\Delta\varphi_1+\Delta\varphi_2)}\hat{a}_3^\dagger + e^{i(\Delta\varphi_1+\Delta\varphi_2')}\hat{a}_2^\dagger - e^{i(\Delta\varphi_2+\Delta\varphi_1')}\hat{a}_1^\dagger + e^{i(\Delta\varphi_1'+\Delta\varphi_2')}\hat{a}_0^\dagger\}|vac\rangle \end{pmatrix}, \tag{10}$$

while the processed output is given by

$$\hat{T}_{\mathrm{MZI}}^{(50:50)}\hat{T}_{\mathrm{128ps}}^{(\mathrm{delay})}\hat{T}_{\mathrm{MZI}}^{(50:50)}\hat{T}_{\mathrm{64ps}}^{(\mathrm{delay})}\hat{T}_{\mathrm{MZI}}^{(50:50)}\begin{pmatrix} 0 \\ \frac{1}{2}\{e^{i(\Delta\varphi_1+\Delta\varphi_2)}\hat{a}_3^\dagger + e^{i(\Delta\varphi_1+\Delta\varphi_2')}\hat{a}_2^\dagger - e^{i(\Delta\varphi_2+\Delta\varphi_1')}\hat{a}_1^\dagger + e^{i(\Delta\varphi_1'+\Delta\varphi_2')}\hat{a}_0^\dagger\}|vac\rangle \end{pmatrix}. \tag{11}$$

This will result in seven time bins (see Fig. 2 of the main text). The central time bin (corresponding to $\hat{a}_3^\dagger$) is the superposition of the generated 4-level entangled state, i.e.,

$$\frac{1}{2}e^{i(\Delta\varphi_1+\Delta\varphi_2+\Delta\varphi_1'+\Delta\varphi_2')}\{\hat{a}_{3+0}^\dagger + \hat{a}_{2+1}^\dagger + \hat{a}_{1+2}^\dagger + \hat{a}_{0+3}^\dagger\}|vac\rangle, \tag{12}$$

and can be retrieved from port $\mathrm{In_1}$. The indices j and k in $\hat{a}_{j+k}^\dagger$ represent the forward and the backward propagation delay, respectively. The global phase factor $e^{i(\Delta\varphi_1+\Delta\varphi_2+\Delta\varphi_1'+\Delta\varphi_2')}$ indicates that, during forward and backward propagation, all the time bins in $\hat{a}_3^\dagger$ propagate over the same path – which means that they experience both the 64 ps and 128 ps delay only once. This compensates for the arbitrary phase offset per delay for a bias-free interference. However, in the case of three-mode mixing (i.e., the interference modes $\hat{a}_2^\dagger$ and $\hat{a}_4^\dagger$), the forward and backward propagations follow different paths. For instance, for those time bins overlapping at $\hat{a}_2^\dagger$, some go through the 64 ps delay twice, while the remaining go through the 128 ps delay once. This leads

to additional phase issues, meaning that the modes interfering in $\hat{a}_2^\dagger$ and $\hat{a}_4^\dagger$ are not in a defined relative phase. This issue prevented us from performing quantum state tomography measurements based on three mode-mixing.

Supplementary Note 2 - BBM92 QKD protocol

Supplementary Note 2.1 - Beam splitter-based delay scheme

In our experiments, we first generated the photon pairs and then routed them into a beam splitter-based delay scheme, where the signal and idler photons went through either the short or long path (as shown in Supplementary Figure 3). The two-photon state after the delay scheme reads

$$|\Phi\rangle = \frac{1}{2}(|S\rangle_s|S\rangle_i + |S\rangle_s|L\rangle_i + |L\rangle_s|S\rangle_i + |L\rangle_s|L\rangle_i), \tag{13}$$

where $|S\rangle_{s(i)}$ and $|L\rangle_{s(i)}$ denote the short and the long path (respectively) that is traveled by the signal (idler) photon. The two photons were then randomly routed to either the time or the phase basis measurement system. The two-photon state experiencing the time and phase basis scheme is thus given by

$$\begin{aligned}|\Phi'\rangle = \tfrac{1}{4}(&|S,t\rangle_s|S,t\rangle_i + |S,t\rangle_s|S,p\rangle_i + |S,p\rangle_s|S,t\rangle_i + |S,p\rangle_s|S,p\rangle_i \\ &+|S,t\rangle_s|L,t\rangle_i + |S,t\rangle_s|L,p\rangle_i + |S,p\rangle_s|L,t\rangle_i + |S,p\rangle_s|L,p\rangle_i \\ &+|L,t\rangle_s|S,t\rangle_i + |L,t\rangle_s|S,p\rangle_i + |L,p\rangle_s|S,t\rangle_i + |L,p\rangle_s|S,p\rangle_i \\ &+|L,t\rangle_s|L,t\rangle_i + |L,t\rangle_s|L,p\rangle_i + |L,p\rangle_s|L,t\rangle_i + |L,p\rangle_s|L,p\rangle_i).\end{aligned} \tag{14}$$

The state $|S,t\rangle_{s(i)}$ denotes the signal (idler) photon traveling the short path S and subsequentially being routed into the time t basis; $|S,p\rangle_{s(i)}$ denotes the signal (idler) photon traveling the short path S and subsequentially being routed into the phase p basis. Similarly, $|L,t\rangle_{s(i)}$ denotes the signal (idler) photon traveling the long path L and subsequentially being routed into the time t basis, while $|L,p\rangle_{s(i)}$ denotes the signal (idler) photon traveling the long path L and subsequentially being routed into the phase p basis. In our work, we considered the cases where both signal and idler go to the same measurement basis, while the other half of the cases were discarded.

Supplementary Note 2.2 - Implementation of the BBM92 protocol with time-bin entangled ququarts (*d*=4)

The 64 ps spacing of the time bins almost approached the ~52 ps jitter time of the SNSPDs used in our experiment. While such a jitter time can still resolve 4-level qudits, it brings a higher crosstalk from adjacent time bins, yielding an increase of the quantum bit error rate (QBER, see section S4). To address this issue, we implemented external temporal gating, which was experimentally implemented by making use of two intensity modulators (IMs in Supplementary Figure 3), one in the time basis and one in the phase basis measurement setup. A variable optical attenuator (VOA in Fig. S3) was used to introduce losses into the signal and idler photons' channel prior to the beam splitter-based delay scheme.

In the time basis measurement system, the temporal gating was applied to select only modes $|0\rangle$ and $|2\rangle$ for the short path of the beam splitter-based delay scheme, and only modes $|1\rangle$ and $|3\rangle$ for the long path. This is described by

$$\begin{aligned} |S,t\rangle_{s(i)} &\to \frac{1}{\sqrt{2}}(|0\rangle + |2\rangle)_{s(i)}, \\ |L,t\rangle_{s(i)} &\to \frac{1}{\sqrt{2}}(|1\rangle + |3\rangle)_{s(i)}. \end{aligned} \tag{15}$$

The measurement operator of the time basis can be expressed as

$$\widehat{M}_t = \frac{1}{2}(|0_s0_i\rangle\langle 0_s0_i| + |1_s1_i\rangle\langle 1_s1_i| + |2_s2_i\rangle\langle 2_s2_i| + |3_s3_i\rangle\langle 3_s3_i|), \tag{16}$$

which was realized here by temporally post-selecting photon events with 25% efficiency (i.e., 6 dB loss). The loss of the external temporal gating system (i.e., the intensity modulator) was 5 dB for each photon. Considering coincidences between the signal and the idler photons, the overall efficiency of the external gating system was 2.51% (i.e., 16 dB loss). If using SNSPDs with lower jitter time[2] (i.e., < 30 ps), the external temporal gating system can be removed, which leads to an enhancement of the time basis measurement by 16 dB. This results in

$$|S(L),t\rangle_{s(i)} \to \frac{1}{2}(|0\rangle + |1\rangle + |2\rangle + |3\rangle)_{s(i)}. \tag{17}$$

In the phase basis measurement system, we applied external phase modulation to project the signal and idler photons into different phase vectors. The external temporal gating was then used to select the superposition state $|f_n\rangle = \frac{1}{\sqrt{d}}\sum_{k=0}^{d-1} e^{i\frac{2\pi}{d}k\cdot n}|k\rangle$ ($n = 0,1,2,3$), which results in

$$|S(L), p\rangle_{s(i)} \rightarrow \frac{1}{2}(|f_0\rangle + |f_1\rangle + |f_2\rangle + |f_3\rangle)_{s(i)}. \tag{18}$$

The measurement operator of the phase basis is given by

$$\widehat{M}_p = \frac{1}{2}(|f_{0_s}f_{0_i}\rangle\langle f_{0_s}f_{0_i}| + |f_{1_s}f_{3_i}\rangle\langle f_{1_s}f_{3_i}| + |f_{2_s}f_{2_i}\rangle\langle f_{2_s}f_{2_i}| + |f_{3_s}f_{1_i}\rangle\langle f_{3_s}f_{1_i}|), \tag{19}$$

which was realized here by temporally post-selecting photon events with 12.5% efficiency

Supplementary Note 2.3 - Implementation of BBM92 scheme with time-bin entangled qubits (*d*=2)

We implemented a BBM92-like QKD scheme[3] with time-bin entangled qubits to compare the performance of our system with current state-of-the-art implementations. The two mutually unbiased bases for qubits are the time basis $\{|0\rangle, |1\rangle\}$ and the phase basis $\{|f_0\rangle = \frac{1}{\sqrt{2}}(|0\rangle + |1\rangle), |f_1\rangle = \frac{1}{\sqrt{2}}(|0\rangle - |1\rangle)\}$. For this purpose, we generated 2-level photon pairs featuring a time bin spacing of 128 ps, the resolution of which was not affected by the SNSPD jitter time. Still, to make a fair comparison between the QKD schemes based on 2- and 4-level entangled states, we utilized the same experimental setting (i.e., IMs for temporal gating + VOA to introduce losses) also for qubits. This gating only selected the time mode $|0\rangle$ for the short path and the time bin $|1\rangle$ for the long path of the beam splitter-based delay scheme. This is described by

$$\begin{aligned} |S, t\rangle_{s(i)} &\rightarrow |0\rangle_{s(i)}, \\ |L, t\rangle_{s(i)} &\rightarrow |1\rangle_{s(i)}. \end{aligned} \tag{20}$$

Thus, the events measured in the time basis were temporally post-selected from the photon events with 25% efficiency, which means that the overall efficiency of the external temporal gating system is the same as the 4-level case (i.e., 2.51%). In the phase basis measurement setting, we used external phase modulation for photon state projections and external temporal gating to select the superposition states $|f_0\rangle = \frac{1}{\sqrt{2}}(|0\rangle + |1\rangle)$ and $|f_1\rangle = \frac{1}{\sqrt{2}}(|0\rangle - |1\rangle)$.

The comparison between ququart- and qubit-based BBM92 schemes is illustrated in Fig. 4d of the main text. The solid lines represent the cases in which no temporal gating is applied, i.e., in the absence of the two IMs. Blue and orange colors are associated with ququarts and qubits, respectively. The blue star represents measurements acquired for entangled ququarts by replacing the VOA with a 30 km-long fiber system, while still applying the external temporal gating (necessary to resolve 64-ps spaced qudits). Instead, since the time mode spacing of the qubits (128

ps) was much larger than the jitter time of the SNSPDs (~52 ps), we could attain experimental data without recurring to the two IMs. The red star represents measurements acquired for entangled qubits by replacing the VOA with a 30 km-long fiber system where each photon propagates and by removing the two IMs (see section S2.5). The implementation of qubit-based BBM92 QKD without external temporal gating led to an enhancement of the secret key rates (SKRs) by a factor of ~40 compared to the case in which the external temporal gating was applied (see Fig. 4 of the main text). This result agreed with the estimated efficiency of 2.51% for the temporal gating.

Supplementary Note 2.4 - Security bound for the BBM92 protocol with time-bin entangled ququarts

Following standard security definitions[4], the QKD protocol is stated as ε-secure if it is both ε_{sec}-secret and ε_{cor}-correct. The protocol is called ε_{sec}-secret if the joint state of the output secret key (e.g., on Alice's side) and of total information from the adversary (e.g., Eve) is statistically indistinguishable from the ideal output state except from some small probability ε_{sec}. The ideal output state is an output key that is uniformly random (in the key space) and completely independent of the adversary's total information. The protocol is called ε_{cor}-correct if the output secret keys on Alice's and Bob's sides are identical except from some small probability ε_{cor}.

The starting point of the security analysis is to ask how many secret bits X can be extracted from Alice's raw key given Eve's total information on the QKD system, denoted as E. To this end, we use the quantum leftover-hash lemma[5] to bound the secret key length l as

$$l = \max_{\beta \in \left(0, \frac{\varepsilon_{\text{sec}}}{2}\right]} \left\lfloor H_{\min}^{\frac{\varepsilon_{\text{sec}}}{2} - \beta}(X|E) + 4 \log_2 \beta - 2 \right\rfloor, \tag{21}$$

where $H_{\min}^{\frac{\varepsilon_{\text{sec}}}{2} - \beta}$ is the smooth min-entropy of X given E, and β is a constrained optimization parameter (more details can be found in Ref.[5]). Then, we use the entropic uncertainty relations for qudits to bound the smooth min-entropy

$$H_{\min}^{\frac{\varepsilon_{\text{sec}}}{2} - \beta}(X|E) \geq n \left[\log_2 d - H_d\left(e_f + \delta(n, k, \beta)\right)\right] - \text{Leak}_{\text{EC}} - \log_2 \frac{2}{\varepsilon_{\text{cor}}}, \tag{22}$$

with

$$\mathrm{Leak_{EC}} = 1.2nH_d(e_t). \tag{23}$$

In this expression, e_t (e_f) is the QBER of the time (phase) basis, $\mathrm{Leak_{EC}}$ is the information leakages for error correction, $\log_2 2/\varepsilon_{\mathrm{cor}}$ is the number of bits published during error verification, and $H_d(x)$ is the d-level Shannon entropy function: $H_d(x) = -x\log_2(x/(d-1)) - (1-x)\log_2(1-x)$.

The term $\delta(n,k,\beta) = \sqrt{(n+k)(k+1)\ln(2/\beta)/nk^2}$ is the noise due to finite-key statistics of the number of raw keys used for parameter estimation k and the raw keys left for key generation n. It quantifies how the data subset used for parameter estimation well represents the entire dataset. We note that $\delta(n,k,\beta)$ goes closer to zero when both n and k become larger. We thus calculate the lengths l of the keys through the expression

$$l = \max_{\beta\in(0,\varepsilon_{\mathrm{sec}}/4)} \left\lfloor n\left[\log_2 d - H_d\left(e_f + \delta(n,k,\beta)\right)\right] - \mathrm{Leak_{EC}} - \log_2 \frac{8}{\beta^4 \varepsilon_{\mathrm{cor}}} \right\rfloor. \tag{24}$$

In the experiment and simulation presented in the main text, we assume that $\varepsilon_{\mathrm{sec}} = 10^{-9}$ and $\varepsilon_{\mathrm{cor}} = 10^{-12}$. As shown in Fig. 4b and 4c of the main text, each data point represents 10 minutes of acquisition time, leading to an average finite-key statistical noise of 1.32% (we made the QKD experiment run for 5 hours). Fig. 4d of the main text reports the SKR versus the channel loss. Experimental data were acquired with a collection interval ranging from 30 to 90 minutes, while the simulated results are obtained by assuming raw key lengths after 1-hour acquisition.

Supplementary Note 2.5 - Quantum state propagation and BBM92 protocol over an optical fiber link (qubits and ququarts)

We demonstrated that our scheme can be utilized for long-distance quantum communications by implementing the BBM92 protocol with both qubits and ququarts over a 60 km-long link of standard telecommunication fibers between two clients. This arrangement consisted of signal and idler photons propagating through a 30 km-long fiber system, comprised of a 20 km-long single-mode fiber (SMF, 4.5 dB loss, 380 ps·nm^{-1}) and a 10 km-long dispersion-compensating fiber (DCF, 2.9 dB loss, -366 ps·nm^{-1}). The picosecond-spaced nature of the time bins led to considerable dispersive broadening upon propagation through the SMF, causing mode mixing and thus ambiguous post-selection of time-resolved coincidences. The dispersion experienced by the

time bins could be compensated for after propagation through a DCF module. We first validated the robustness of the 4- and 2-level entangled photon states over fiber transmission by measuring quantum interference after propagation. We then extracted the visibilities, which maintained values well above the threshold necessary to violate their respective Bell inequalities (see Supplementary Figure 4). The same 60 km-long fiber link was used to implement the BBM92 protocol, the results of which well fits our simulations (see Fig. 4 of the main text).

Supplementary Note 3 - Characterization of the entanglement source (the spiral waveguide)

The signal and idler photon pairs generated from the spiral waveguide through SFWM follow a thermal distribution and their quantum state can be expressed as[6]

$$|\Psi\rangle = \sum_{n=0}^{+\infty} \left[\frac{\mu^n}{(\mu+1)^{n+1}}\right]^{\frac{1}{2}} |n\rangle_s |n\rangle_i. \tag{25}$$

Assuming a pulsed laser pumping scheme (as in our work), in this expression μ is the mean photon pair per pulse generated by the SFWM process, while n is the number of signal (s) and idler (i) photons generated per pulse. Assuming $\mu \ll 1$, the mean photon pair per pulse quadratically scales with the peak pump power P as $\mu(P) = aP^2$, where the coefficient a expresses the photon pair generation efficiency at a given pump peak power. In contrast, the generation of uncorrelated photons from Raman scattering scales linearly with the peak pump power. The probability of detecting any noise photons per pulse is given by $\xi(P) = bP + c$, where the coefficient b expresses the generation efficiency of Raman photons produced per pulse and c is a constant noise fraction that depends on the dark counts of the single-photon detectors. Assuming that the total collection efficiency η and the noise level ξ for signal and idler channel are the same, the two-photon coincidence probability results in

$$P_{co} = \eta^2\mu(1+\mu) + (\xi + \eta\mu)^2, \tag{26}$$

with $P_{ac} = (\xi + \eta\mu)^2$ being the accidental probability, governed by uncorrelated Raman photons and multi-photon events.

Based on Eq. (26), we can measure two-photon coincidences and accidentals at different pump powers, so as to estimate the coefficients a and b, and thus characterize the spiral waveguide. The mode-locked laser used to pump the spiral, centered at 1,556.15 nm and featuring a repetition rate of 250 MHz, was spectrally filtered to ~5 ps pulse duration. The total collection efficiency per

photon is $\eta = 8.8\%$, which includes the losses of the spiral waveguide (coupling losses, ~2.2 dB), of the programmable filter (~5 dB), of other fiber components (~2 dB), and of the SNSPDs (~0.7 dB). Supplementary Figure 5a shows the experimental results of the coincidence-to-accidental ratio (CAR) values at different peak pump powers, while Supplementary Figure 5b shows the CAR values versus the coincidence counts. By fitting the data points (respectively, solid red lines and black circles in Supplementary Figure 5) based on Eq. (26), we obtain $a = 1.527\times10^{-4}$ W^{-2} and $b = 1.722\times10^{-3}$ W^{-1}. The CAR could achieve a value of 50 for the low pump power regime, where the Raman noise is less detrimental. However, in our experiment, to balance the Raman noise and the photon pair generation rate from the SFWM process, we selected and operated at a CAR value of 20 for quantum interference and QKD implementations. This required pumping the spiral waveguide at a relatively high peak power of ~6.5 W. We note that the estimated generation efficiency of photon pairs produced by SFWM is orders of magnitude lower than for other on-chip waveguide sources[7–9]. The quality of the time-bin entangled qudits can be further improved by using on-chip two-photon sources with higher brightness and lower noise. The brightness of our spiral waveguide is 1.018×10^{-12} pairs/(mW^2·GHz) per pulse. State-of-the-art on-chip waveguide sources made from other materials (e.g., silicon) report higher brightness and CAR values as compared to our spiral waveguide. For instance, in Ref.[8] a brightness of 1.535×10^{-9} pairs/(mW^2·GHz) per pulse and a CAR of 400 are achieved, while in Ref.[7] the registered values amount to 2.993×10^{-5} pairs/(mW^2·GHz) per pulse and a CAR of 1633, respectively. Such high efficiencies could enable ~MHz levels of photon pair generation rates under significantly lower pump peak powers (a few mW), ultimately reducing Raman scattering effects and increasing the CAR. We note that, despite the relatively low performance of the spiral waveguide, our scheme could still achieve good results in terms of entangled qudits' quantum interference, quantum state tomography, and BBM92-like QKD protocol. This was possible due to the ability of leveraging the on-chip cascade interferometric system.

Based on the coefficients a and b obtained from the source characterization, we simulated the CAR values at different peak pump powers considering the total collection efficiencies associated to entangled ququart-based QKD measurements in the time and phase bases ($\eta_t = 1.50\%$ and $\eta_p = 0.026\%$, respectively). The results from simulations are shown in Supplementary Figure 6a. At a pump peak power of ~6.5 W, CAR values are still maintained at around 20 for both time and phase basis measurements (blue and red curves, respectively). In Supplementary Figure 6b we report the

simulated QBER versus the peak power for the time and phase bases (blue and red curves, respectively). Simulations match with the QBERs measured in our work for the time and phase bases (blue and red triangles, respectively). Finally, we calculated the estimated SKR by considering a 4-fold pump burst with a repetition rate of 1 GHz, based on the same collection efficiency and QBER shown before. The blue curve in Supplementary Figure 6c illustrates the simulated results, which indicate that, when the spiral waveguide is pumped with a 4-fold burst at ~6.5 W peak power, the SKR reaches a maximum of ~2.5 kbit/s. For our QKD demonstration, we chose the pump power based on these simulations. The black triangle in Supplementary Figure S6c shows the experimental output obtained for the SKR by using finite-key estimation, which results lower than the maximum obtained in the simulation curve.

Supplementary Note 4 - Characterization of the jitter time of the SNSPDs for the detection of time-bin entangled ququarts

The 64 ps spacing of the time bins almost approached the ~52 ps jitter time of the SNSPDs used in our experiment. As shown in Supplementary Figure 7a, the single-photon histogram indicates that, even though the detector jitter time can still resolve ququarts, it brings crosstalk from adjacent time bins. The shadow regions are Gaussian functions obtained from the photon counting histogram of a single pulse, which has a full width at half maximum (FWHM) of ~52 ps. The crosstalk can also be visualized by the coincidence matrix among all time bins of the entangled ququarts, as shown in Supplementary Figure 7b, which yields a QBER of 23.40%. Such value is beyond the QBER threshold for 4-level QKD. To address this issue, we implemented external temporal gating in the time basis measurement system that selected only modes $|0\rangle$ and $|2\rangle$ for the short path of the beam splitter-based delay scheme, and only modes $|1\rangle$ and $|3\rangle$ for the long path, as shown in Supplementary Figure 7c and 7d, respectively. The external temporal gating system enabled a time-bin spacing of 128 ps, which can be fully resolved by the SNSPDs. The coincidence matrix among all time bins of the entangled ququarts with the external temporal gating system is shown in Supplementary Figure 7e. The overall QBER is thus reduced to ~11%. Such residual QBER originates from the source noise. Furthermore, as illustrated in Supplementary Figure 8, we simulated the estimated QBER of our 64 ps-spaced entangled ququarts versus the jitter time of SNSPDs. We note that if SNSPDs with lower jitter time[2] (i.e., < 30 ps) could be used, the detection crosstalk of the adjacent time bins could be eliminated. This would make it possible to remove the

external temporal gating system, and thus achieve the SKR for 4-level QKD predicted by the solid blue line shown in Fig. 4d of the main text (named there as “without gating curve”, w/o in Fig. 4 of the main text).

Figures (Supplementary Information)

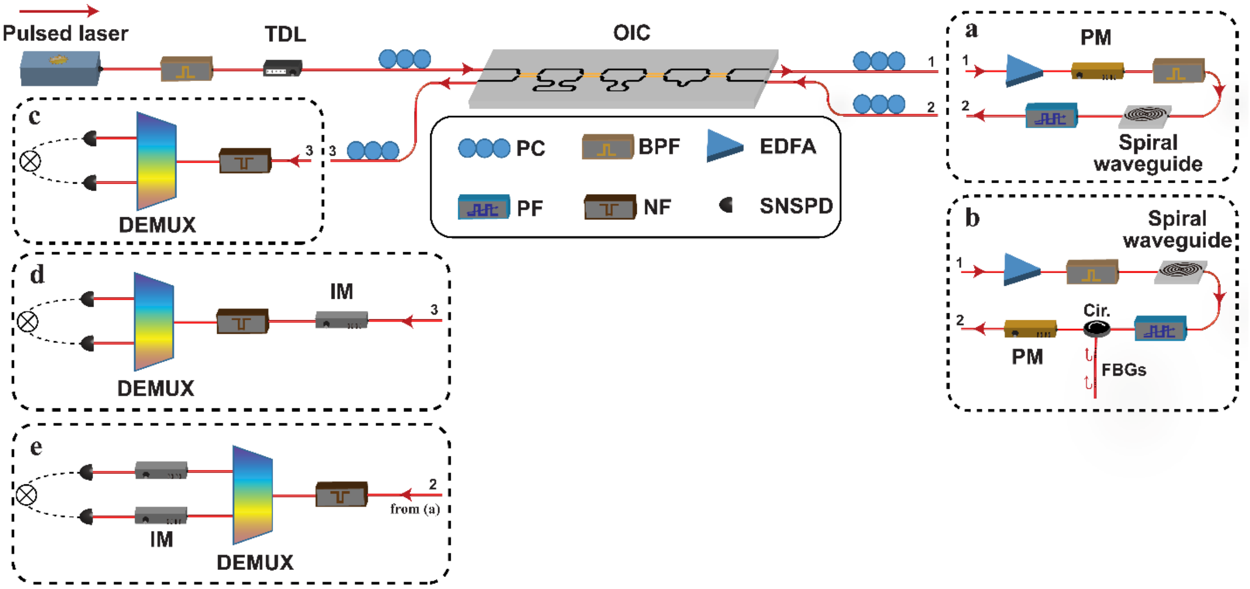

Supplementary Figure 1. Experimental modules for quantum state processing. Modular combination used for: **a) + d)** quantum interference (i.e., for 4- and 8-level entangled states), **b) + c)** quantum state tomography, and **a) + e)** joint temporal distribution measurement. TDL: tunable delay line, OIC: on-chip interferometer cascade, PM: phase modulator, IM: intensity modulator, PC: polarization controller, BPF: band pass filter, EDFA: erbium-doped fiber amplifier, PF: programmable filter, NF: notch filter, Cir: circulator, FBG: fiber Bragg grating, DEMUX: demultiplexer, SNSPD: superconducting nanowire single-photon detector.

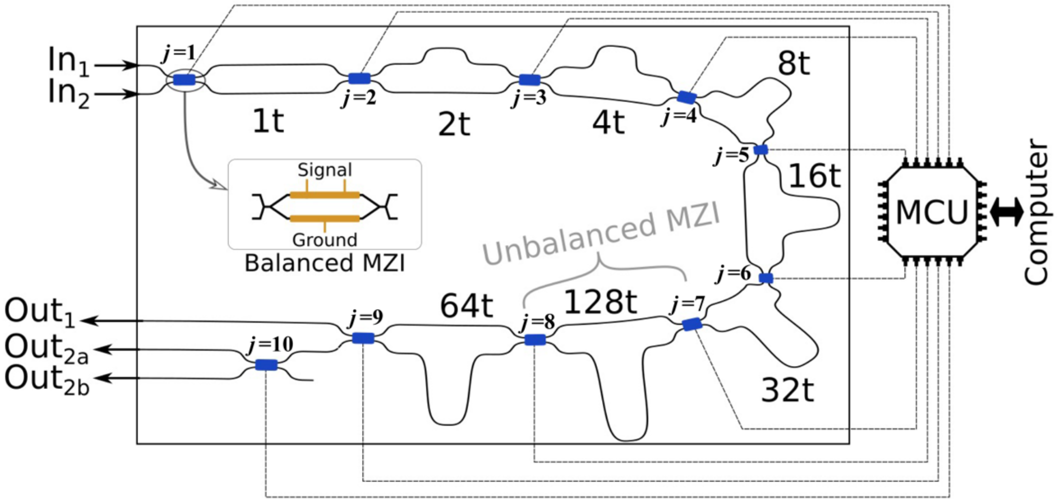

Supplementary Figure 2. Schematic representation of the on-chip interferometer cascade (OIC). The chip consists of a concatenation of balanced and unbalanced Mach-Zehnder interferometers (MZIs). The OIC can be accessed through two input and three output fiber-coupled waveguides. Electric heaters are used to control both the phase of the balanced MZIs and the power ratio entering each arm of the following unbalanced MZI. The balanced MZIs consist of two phase-tunable waveguides of the same length within two 50:50 waveguide couplers (inset). The maximum accessible number of time bins in our platform is 256 with a time bin spacing of 1 ps.

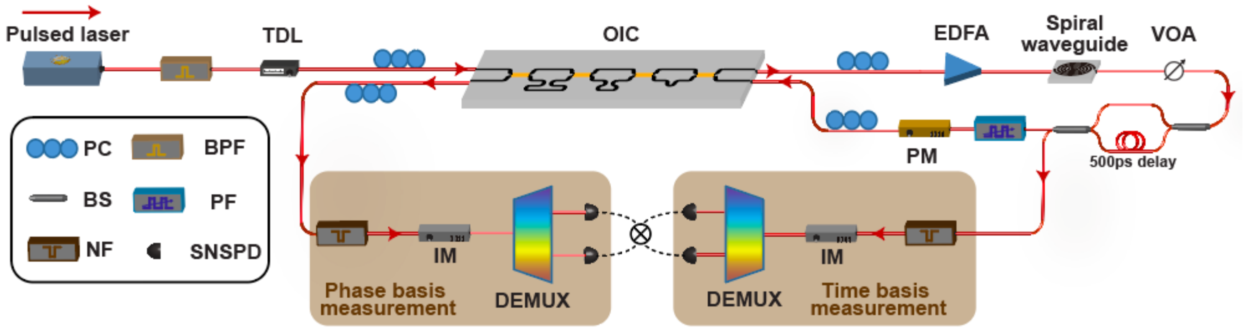

Supplementary Figure 3. Setup for BBM92-like QKD based on time-bin entangled states. Following the photon pair generation stage, a variable optical attenuator was used to introduce losses into the signal and idler photons' channel prior to the fiber-based delay system in order to simulate transmission losses. A fiber-based delay system was used to reproduce the random choice of the mutually unbiased bases by Alice and Bob. Photons are randomly directed into either the time- or phase-measurement setups through two 50:50 beam splitters, either at the same time or with a 500 ps temporal delay. Time basis measurements were achieved by applying temporal gating to photons, while phase basis measurements were carried out by applying electro-optic phase modulation and then temporal gating on the time bin superposition. Such temporal gating was implemented by making use of two intensity modulators, one in the time basis and one in the phase basis measurement setup. The sifted keys were collected from the coincidence events obtained from the time basis measurements, while the security of the system was monitored through the bit error rates from both the time and phase basis measurements. TDL: tunable delay line, OIC: on-chip interferometer cascade, EDFA: erbium-doped fiber amplifier, VOA: variable optical attenuator, PM: phase modulator, IM: intensity modulator, DEMUX: demultiplexer, PC: polarization controller, BPF: band pass filter, BS: beam splitter, PF: programmable filter, NF: notch filter, SNSPD: superconducting nanowire single-photon detector.

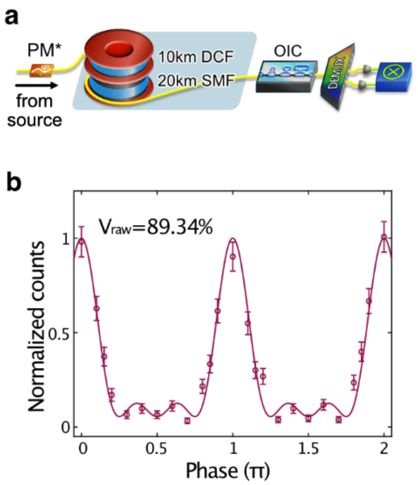

Supplementary Figure 4. Propagation of the time-bin entangled ququarts. a) Signal and idler photons propagate each over a 20 km single-mode fiber (SMF) and 10 km dispersion-compensating fiber (DCF). This mimics two users who are separated by 60 km. The presented setup was also used for quantum interference measurements after photon propagation. The position of the phase modulator (PM*) is indicated in the setup for completeness. **b)** Measured quantum interferences for 4-level (qudit) entangled photon pairs with a raw visibility of 89.34%±2.55%. Error bars are estimated using Poisson statistics.

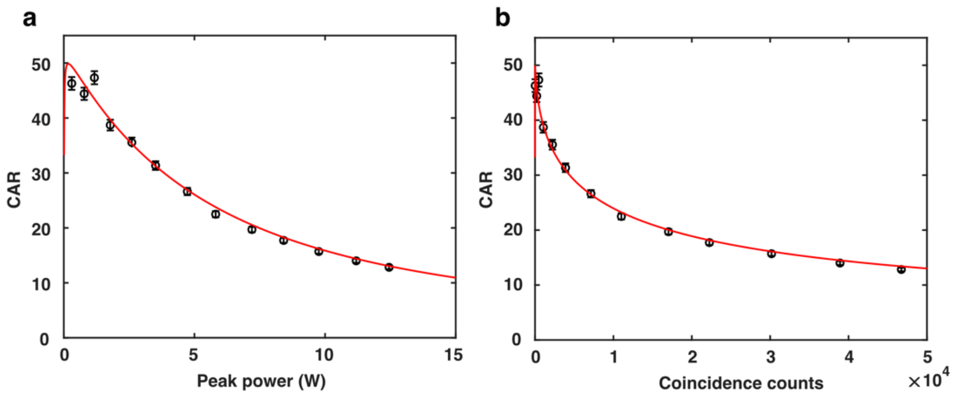

Supplementary Figure 5. Characterization of the spiral waveguide. a) CAR values at different peak pump powers. **b)** CAR values versus the coincidence counts. Black circles are experimental data points, while the two red curves are fitted based on Eq. (26). Error bars are estimated using Poisson statistics.

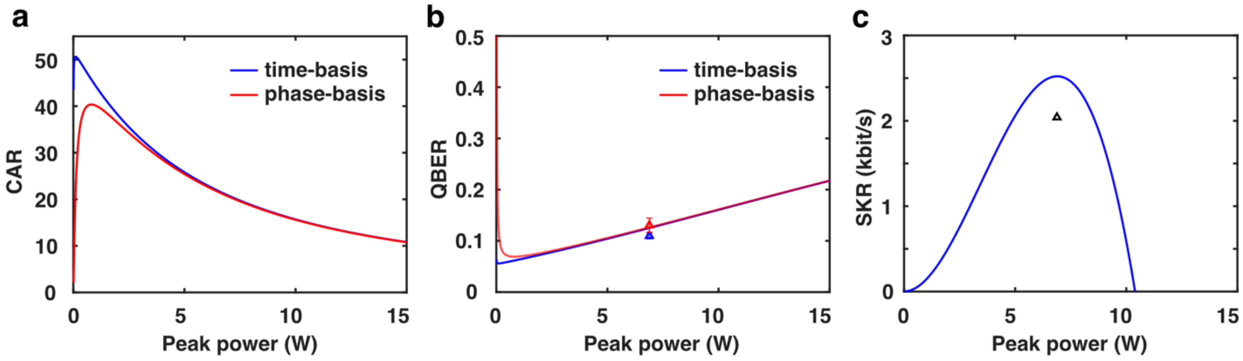

Supplementary Figure 6. Simulations of the time and phase bases measurement according to the source characterization. a) CAR values at different peak pump powers considering the total collection efficiencies for the time ($\eta_t = 1.50\%$) and phase ($\eta_p = 0.026\%$) bases measurement. **b)** Simulated QBER versus the pump peak power for the time and phase bases. Blue and red triangles are experimental data. **c)** Estimated secret key rate. The black triangle represents the experimental data. Error bars are estimated using Poisson statistics.

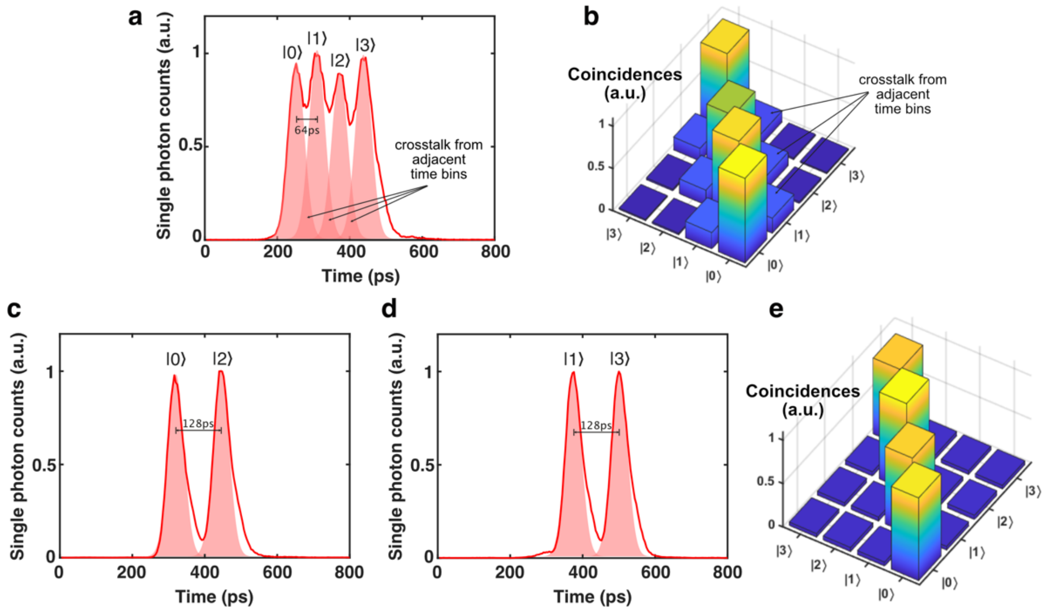

Supplementary Figure 7. Analysis of the jitter time of the SNSPDs on the time-bin qudits' resolution. a) Single-photon histogram of the 4-level qudits. The shadow regions are Gaussian functions obtained from the photon counting histogram of a single pulse, which has a full width at half maximum (FWHM) of ~52 ps. **b)** Coincidence matrix among all time bins of the 4-level qudits. The post-selection time window was 44 ps. **c,d)** Single-photon histograms of the 4-level qudits after applying an external temporal gating. **e)** Coincidence matrix among all time bins of the entangled ququarts, obtained by using the external temporal gating. The post-selection time window was 100 ps.

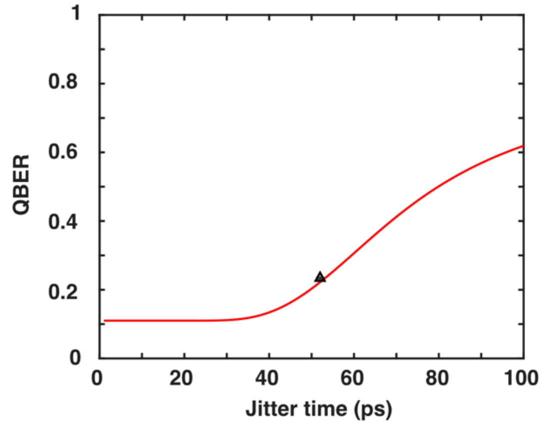

Supplementary Figure 8. Simulations of the QBER of 64 ps-spaced entangled ququarts versus the jitter time of SNSPDs. The black triangle is the experimental data obtained without external temporal gating. The post-selection time window was fixed at 44 ps. Error bar is estimated using Poisson statistics.

References

1. Fischer, B. *et al.* Autonomous on-chip interferometry for reconfigurable optical waveform generation. *Optica* **8**, 1268–1276 (2021).
2. Esmaeil Zadeh, I. *et al.* Efficient single-photon detection with 7.7 ps time resolution for photon-correlation measurements. *ACS Photonics* **7**, 1780–1787 (2020).
3. Bennett, C. H., Brassard, G. & Mermin, N. D. Quantum cryptography without Bell's theorem. *Phys. Rev. Lett.* **68**, 557–559 (1992).
4. Müller-Quade, J. & Renner, R. Composability in quantum cryptography. *New J. Phys.* **11**, 085006 (2009).
5. Tomamichel, M. & Hayashi, M. A Hierarchy of information quantities for finite block length analysis of quantum tasks. *IEEE Trans. Inf. Theory* **59**, 7693–7710 (2013).
6. Zhu, F., Tyler, M., Valencia, N. H., Malik, M. & Leach, J. Is high-dimensional photonic entanglement robust to noise? *AVS Quantum Sci.* **3**, 011401 (2021).
7. Jin, M. *et al.* Photon-pair generation in a heterogeneous nanophotonic chip. *ACS Photonics* **10**, 1962–1968 (2023).
8. Harada, K. *et al.* Frequency and polarization characteristics of correlated photon-pair generation using a silicon wire waveguide. *IEEE J. Sel. Top. Quantum Electron.* **16**, 325–331 (2010).
9. Paesani, S. *et al.* Near-ideal spontaneous photon sources in silicon quantum photonics. *Nat. Commun.* **11**, 2505 (2020).
